# Mapping memory binding onto the connectome's temporal dynamics: toward a combined biomarker for Alzheimer's disease

**DOI:** 10.3389/fnhum.2014.00237

**Published:** 2014-04-22

**Authors:** Agustin Ibanez, Mario A. Parra

**Affiliations:** ^1^Laboratory of Experimental Psychology and Neuroscience (LPEN), Institute of Cognitive Neurology (INECO), Favaloro UniversityBuenos Aires, Argentina; ^2^Laboratory of Cognitive and Social Neuroscience, UDP-INECO Foundation Core on Neuroscience, Diego Portales UniversitySantiago, Chile; ^3^National Scientific and Technical Research CouncilBuenos Aires, Argentina; ^4^Universidad Autónoma del CaribeBarranquilla, Colombia; ^5^Psychology, Human Cognitive Neuroscience and Centre for Cognitive Ageing and Cognitive Epidemiology, University of EdinburghEdinburgh, UK; ^6^Alzheimer Scotland Dementia Research Centre, Scottish Dementia Clinical Research NetworkEdinburgh, UK

**Keywords:** Alzheimer disease, connectome, EEG/MEG, biomarkers, early detection, mild cognitive impairment

The classical amyloid cascade hypothesis of Alzheimer's disease (AD) has driven research and clinical practice for several decades. It states that the deposition of the amyloid-β peptide in the brain parenchyma initiates a sequence of events that ultimately lead to atrophy and AD dementia. This proposal stimulated the study of specific brain regions mapped along the neurodegeneration sequence (e.g., hippocampus) and their associated impaired functions (e.g., episodic memory). Although anticipated by Mesulam more than 20 years ago (e.g., Mesulam and Asuncion Moran, 1987), it was not until recently that this view has started to change, largely due to the disappointing results of trials relying on the beta-amyloid cascade hypothesis and its variants, e.g., the synaptic beta-amyloid hypothesis.

A new approach based on neuroplasticity of neural networks has shifted the attention from one region to the orchestration of several brain hubs. The connectivity account would play an important role in revealing the transneural spread of misfolded proteins through neural networks in neurodegenerative disease (Pievani et al., 2011; Ibanez and Manes, 2012), and specifically in AD (Raj et al., 2012). In line with this view, the metabolism hypothesis (MH) has been proposed, which suggests that changes in the default mode network (DMN, the ongoing low-frequency fluctuations during resting state between the anterior and posterior cingulate cortex as well as the precuneus) stimulate an activity-dependent or metabolism-dependent cascade that promotes the development of the AD pathology (Buckner et al., 2005). Notably, hyperactive neurons are observed near amyloid plaques in animal models (Busche et al., 2008) and in humans, connectivity hubs overlap the anatomy of A-β deposition (Buckner et al., 2009). Abnormal DMN activity discriminates between Mild Cognitive Impairment (MCI), AD and controls (Rombouts et al., 2005; Petrella et al., 2011; Seo et al., 2013; Wang et al., 2013b), and predicts AD conversion (Pievani et al., 2011). Thus, default connectivity seems to be a promising approach to reveal novel mechanisms leading to AD.

However, the DMN seems to be affected by many other diseases (Sonuga-Barke and Castellanos, 2007; Whitfield-Gabrieli and Ford, 2012). Moreover, although the altered DMN might interrupt or affect the brain dynamic in AD patients, it actually reflects a resting state activity unlikely to explain, on its own, profiles of cognitive decline in AD. The combined analysis of brain connectivity associated with specific cognitive processes affected by AD early in its course with resting DMN is an unexplored area that could help overcome these limitations. This approach may reveal markers for the early or even preclinical detection of neurocognitive impairments in AD. A potential strategy would be to assess the neural connectivity associated with episodic memory tasks (Wang et al., 2013a). However, these tasks have only detected AD-related changes in its prodromal or clinical stages (Fields et al., 2011). A recently developed methodology, namely short-term memory binding (STMB, Parra et al., 2009, 2010, 2011), is intrinsically related to brain networks activation, and appears to be more promising for the preclinical detection of AD. Binding functions, as originally investigated in perception, require a large-scale network integration mechanism (Varela et al., 2001). In AD research, emerging evidence suggests that binding impairments occur at the short term memory level. STMB is a cognitive function responsible for retaining, on a temporary basis, intra-item features thus contributing to the formation of objects' identity. It has been recently assessed with a change detection tasks, which ask participants to judge whether the content of two consecutive arrays of shapes, colors or shape-color combinations is the same or different. STMB is impaired early in AD (Parra et al., 2009) and also in preclinical familial AD (Parra et al., 2010, 2011), preserved in healthy aging (Brockmole et al., 2008), and declines earlier in AD than in other dementias (Della Sala et al., 2012). Moreover, STMB seems to be indexing a pre-hippocampal phase of AD (Reiman et al., 2010) and recruits other regions (Parra et al., 2014). Thus, a combination of both neural (DMN) and cognitive (STMB) integration processes may contribute an early and specific marker of AD progression. A new research agenda linking resting brain dynamics (DMN) with an active task which (1) relies on neural network integration and (2) is highly specific and sensitive for AD, would represent a powerful approach to the early detection of AD. Importantly, the resting and active connectivity measures drawn from this novel approach can be tracked both with neuroimaging (fMRI) and electromagnetic methods (EEG and MEG).

STMB tasks are well suited to be tracked with EEG or MEG measurement (Luria and Vogel, 2011; Wilson et al., 2012). Typically, the temporal dynamic of the integrative functions assessed by this task would be in the order of milliseconds. These techniques can capture the evoked responses and their neural connections during the whole process of STMB. This task also provides several advantages for EEG/MEG procedures such as high number of trials, stimuli-evoked activity pre and post memory binding process, temporal sequencing, and categories with different levels of difficulty.

Notwithstanding the adequacy of the task's parameters for EEG/MEG recordings, the question of why techniques with low spatial resolution should be considered instead of neuroimaging methods stands out. Several factors support this selection. First, high-density electroencephalography (hd-EEG) and other electromagnetic techniques permit an easy, low-cost, non-invasive, and accessible approach for large-scale multisite studies around the world. Second, high density EEG/MEG technologies have provided an increased spatial resolution of fine temporal dynamics both at the analytical (mathematical methods) and technical (high number of channels, photometry methods, and individual MRI co-recordings) level, making them more suitable to investigate AD-related changes. Third, EEG/MEG techniques have proven useful for characterizing AD and also for detecting changes in preclinical familial AD and MCI (Jackson and Snyder, 2008; Stam, 2010). For example, source EEG functional network disruption in AD is associated with cognitive decline (Gianotti et al., 2007; Ishii et al., 2010) (see (Kurimoto et al., 2008; Hsiao et al., 2013) for similar results in MCI), APOE genotype (Canuet et al., 2012) and differentiates between other dementias (Babiloni et al., 2004). Moreover, loss of interregional synchronization between different functional brain regions also reflects cognitive decline in AD (De Haan et al., 2012). Furthermore, the EEG/MEG based connectivity analysis (EMCA) can also be used to track the effect of medication on AD (Babiloni et al., 2006; Gianotti et al., 2008).

There are other direct advantages of using EMCA. The theoretical frame of interdependence between spontaneous and evoked neuroelectric oscillations in terms of frequency and phase reset has been forwarded earlier (e.g., by Basar' group in the eighties). Nevertheless, over the last 10 years, a real increase in technical and mathematical sophistication in EMCA has produced new research possibilities with practical applications (Basar et al., 2013; Larson-Prior et al., 2013). Regarding brain global properties, current graph theoretical network studies of the brain have shown a self-organized small-world network characterized by a combination of focal connectivity and long-distance connections. Graph theory is one of the most powerful forms of connectivity analysis for AD (Pievani et al., 2011; Tijms et al., 2013), and it can be correctly implemented with EEG/MEG signals (e.g., Stam and Van Straaten, 2012; Barttfeld et al., 2013). Regarding time dynamics, different networks are orchestrated in our brain in time windows of milliseconds and the connectivity within and between them is not a static process. Our brain has rapid rhythms that allow for communication between different regions at several frequencies. The high time-resolution of intracranial signals from EEG sources can be quantified by coherence and phase synchronization, two methods that have proved informative in AD (Czigler et al., 2006; Knyazeva et al., 2012). Moreover, recent methods provide a better characterization of the physiological signal with better spatial location and provide solutions for classical problems of volume conduction (Pascual-Marqui et al., 2011). They also permit the comparison of spontaneous and stimulus-induced activations and the identification of commonalities between them (Lehmann et al., 2010). Moreover, oscillatory neuronal dynamics in the human brain using connectivity analysis of source estimated event-related synchronization at different frequencies is now an available method (Ishii et al., 2013). Transient momentary events (e.g., thoughts) in electromagnetic signals might be incorporated in temporal chunks of processing (10–100 ms) as quasi-stable brain states (Lehmann et al., 2006). In brief, EMCA can track the brain dynamic of rapid fluctuations (Barttfeld et al., 2014) and also of transient activity during very short periods, especially those supporting binding or transient integration.

Thus, assessing the combination of basal resting state influences together with the ongoing activity during a task and its evoked neural response may allow for investigation and characterization of preclinical AD-related changes in brain dynamics. This novel approach offers new possibilities to better understand the cognitive binding problem in the course of AD as well as the dynamics of cortical integration. This research proposal also requires tackling several methodological and empirical challenges. Although promising, current methods for combining connectivity measures during ongoing activity and evoked responses do not yet fully capture the single trial dynamics. The potential use of connectivity metrics as predictor of patients' clinical outcome is not well understood at present. No single study using EMCA to assess AD or MCI patients has combined active and resting recordings and the analysis of source connectivity using individual MRI. We believe this combined approach would disclose unknown AD mechanisms. The research growth in these domains of cognitive neuroscience will offer support to key strategies such as combining STMB and EMCA to provide a neurocognitive marker for the preclinical detection of AD. In support to this proposal, recent neuroimaging studies carried out in cases of preclinical familial AD have revealed a temporal proximity between the onset or appearance of STMB deficits and amyloid-β deposition. By the average age at which amyloid-β depositions reach a plateau (Fleisher et al., 2012), STMB deficits become detectable behaviorally (Parra et al., 2010). It is worth noticing that these mutation carriers would be otherwise completely asymptomatic. This evidence warrants investigation of the hypothesis of a link between connectivity problems as assessed by STMB and EMCA and neurodegenerative changes in AD. Such research would shed further light into the link, or lack thereof, between amyloid changes, cognition and AD.

## Conflict of interest statement

The authors declare that the research was conducted in the absence of any commercial or financial relationships that could be construed as a potential conflict of interest.
